# Supplementary material for: A FtsZ cis disassembly element acts in Z-ring assembly during bacterial cell division
Source: Nat Commun. 2025 Jun 4;16:5194. doi: 10.1038/s41467-025-60517-7 (PMC12137876; doi:10.1038/s41467-025-60517-7)
Supplement: Supplementary file 1 — Supplementary Information [file 41467_2025_60517_MOESM1_ESM.pdf]

Supplementary Information for

A FtsZ *cis* disassembly element acts in Z-ring assembly during bacterial cell division

Huijia Yin[1,2]†, Yang Liu[1]†, Ying Zhao[1]†, Pengyue Chen[1] and Zengyi Chang[1,3]*

[1]State Key Laboratory of Gene Function and Modulation Research, School of Life Sciences, Peking University, Beijing 100871, P.R. China

[2]Institute of Geriatrics, National Clinical Research Center for Geriatric Diseases, Second Medical Center of Chinese PLA General Hospital, Beijing 100853, P.R. China

[3]Center for Protein Science, Peking University, Beijing 100871, P.R. China

†These authors contributed equally to this work

*Correspondence: changzy@pku.edu.cn

This PDF file includes:

Supplementary Figures 1 to 8
Supplementary Tables 1 to 6

Supplementary Fig. 1

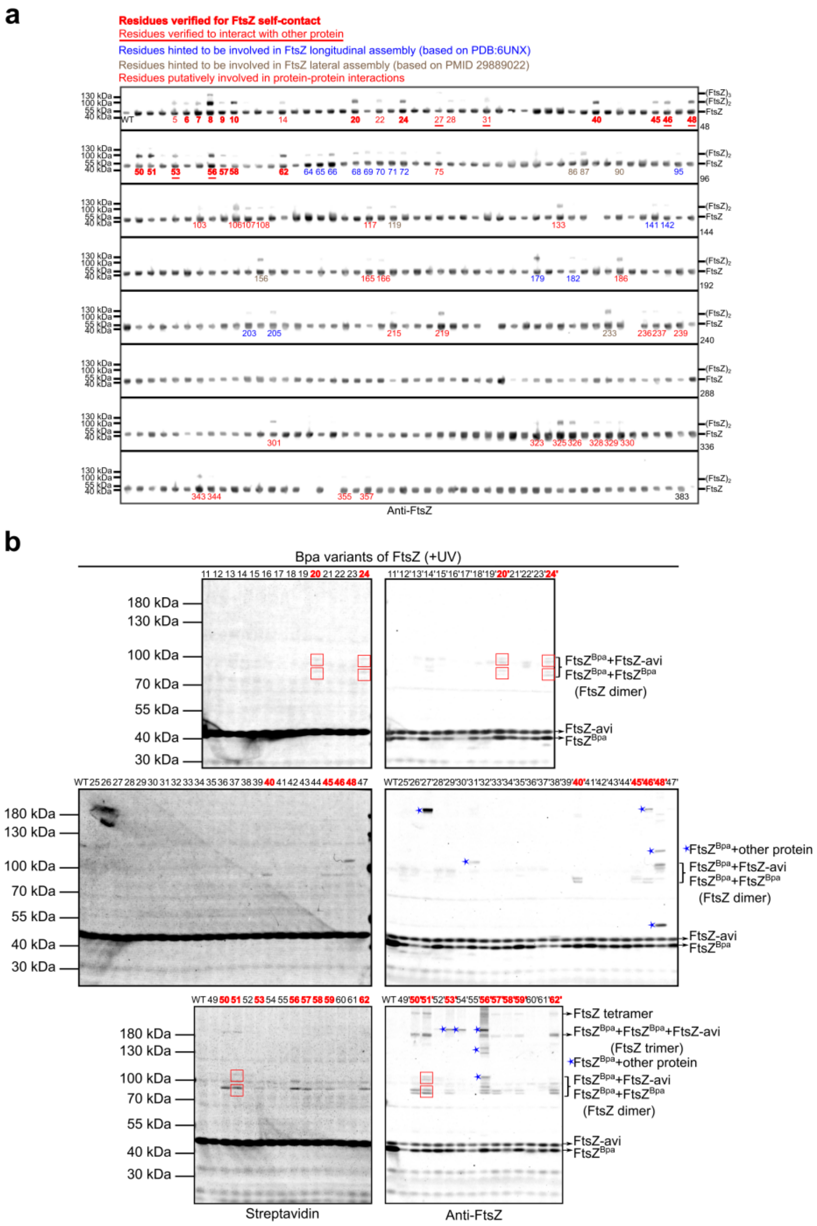

Supplementary Figure 1. The identification of all FtsZ self-assembly residues involved in Z-ring organizing.

a Immunoblots showing photocrosslinked products in all 383 Bpa variants after high-throughput horizontal SDS-PAGE analyses, and probed with polyclonal antibodies against FtsZ. The residues that formed crosslinked products are categorized into the indicated five groups; of note, residues 323-357 were verified to be involved in self-contacts though the data are not shown here. “Residues hinted to be involved in FtsZ longitudinal assembly” were defined as interface residues located within 4.5 Å to a neighboring subunit; “Residues hinted to be involved in FtsZ lateral assembly” were defined as residues located near one previously reported as mediating lateral assembly. On the right are indicated FtsZ protein forms, and on the left are molecular weight marker positions. Source data are provided as in the Source Data file. **b** Immunoblots showing the photocrosslinked products of selected residues in the N-domain after regular vertical SDS-PAGE analyses, and probed with streptavidin (left) or an anti-FtsZ antibody (right). The two photocrosslinked products, indicating a particular residue (e.g., G20, F24 or K51) are putatively involved in two different surfaces, and are each labeled by a red square. On the right of the gels are indicated protein band forms, and molecular weight marker positions are indicated on the left. Blue asterisks indicate photocrosslinked products formed between $FtsZ^{Bpa}$ and other proteins. Source data are provided in the Source Data file.

Supplementary Fig. 2

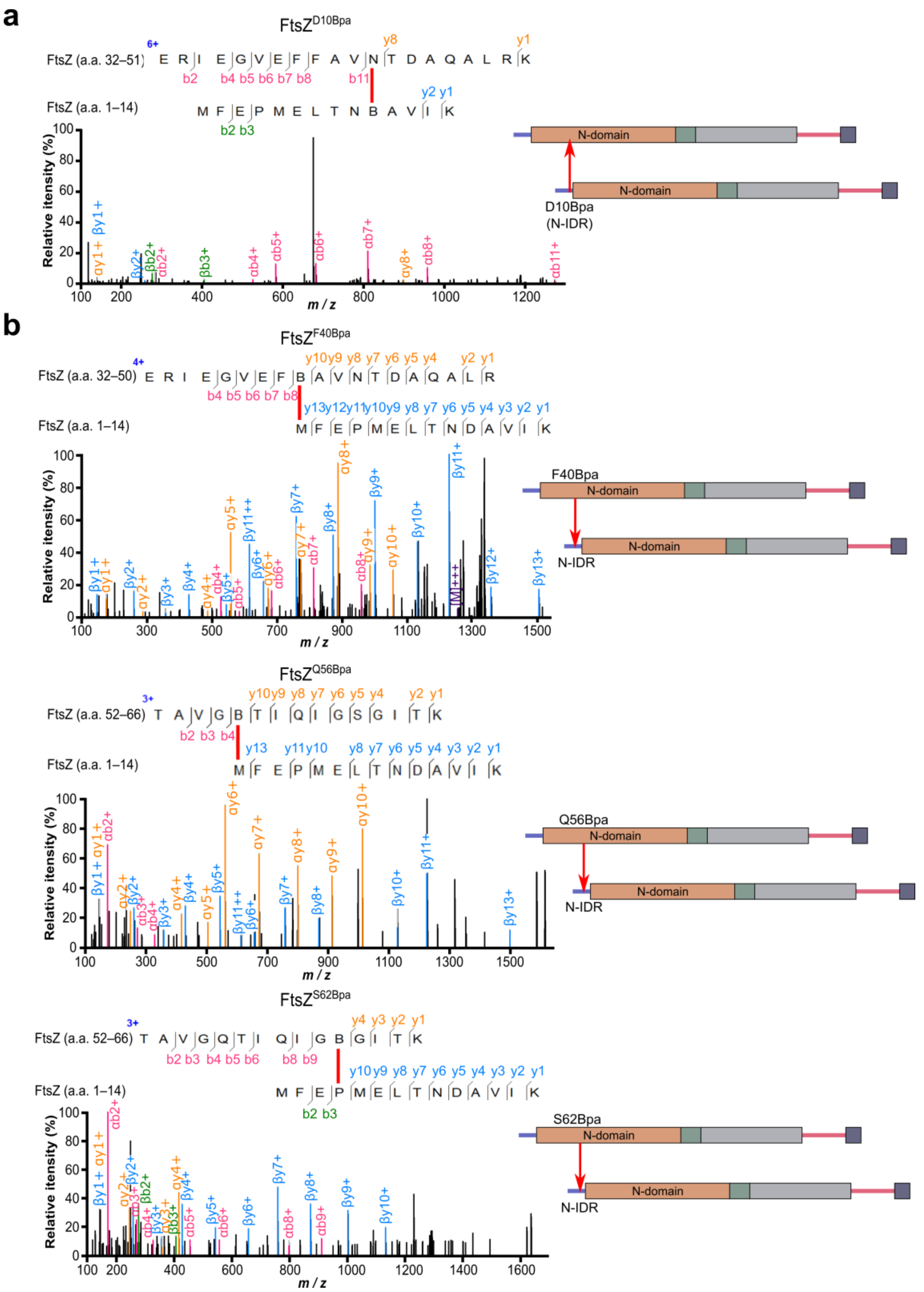

Supplementary Figure 2. The N-IDR interacts with the N-domain for FtsZ self-assembly. MS/MS spectra and schematics showing the identification of the peptide fragment crosslinked with D10Bpa (**a**) or with F40Bpa, Q56Bpa, and S62Bpa (**b**); B, Bpa. Source data are provided in the Source Data file.

Supplementary Fig. 3

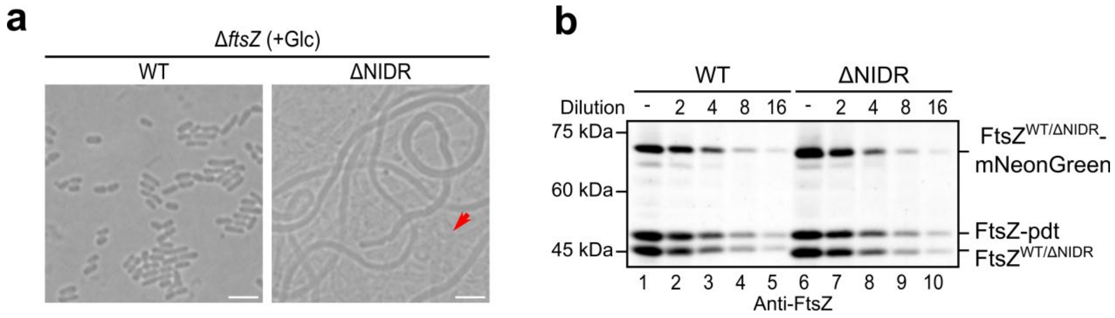

Supplementary Figure 3. N-IDR removal from FtsZ prevents cell division.

a Brightfield micrographs showing residual cells expressing $FtsZ^{\Delta NIDR}$; the red arrow indicates dead cell debris; scale bar = 5 µm. **b** Immunoblot showing that $FtsZ^{\Delta NIDR}$ was expressed at levels comparable to wild-type FtsZ. Source data are provided in the Source Data file.

Supplementary Fig. 4

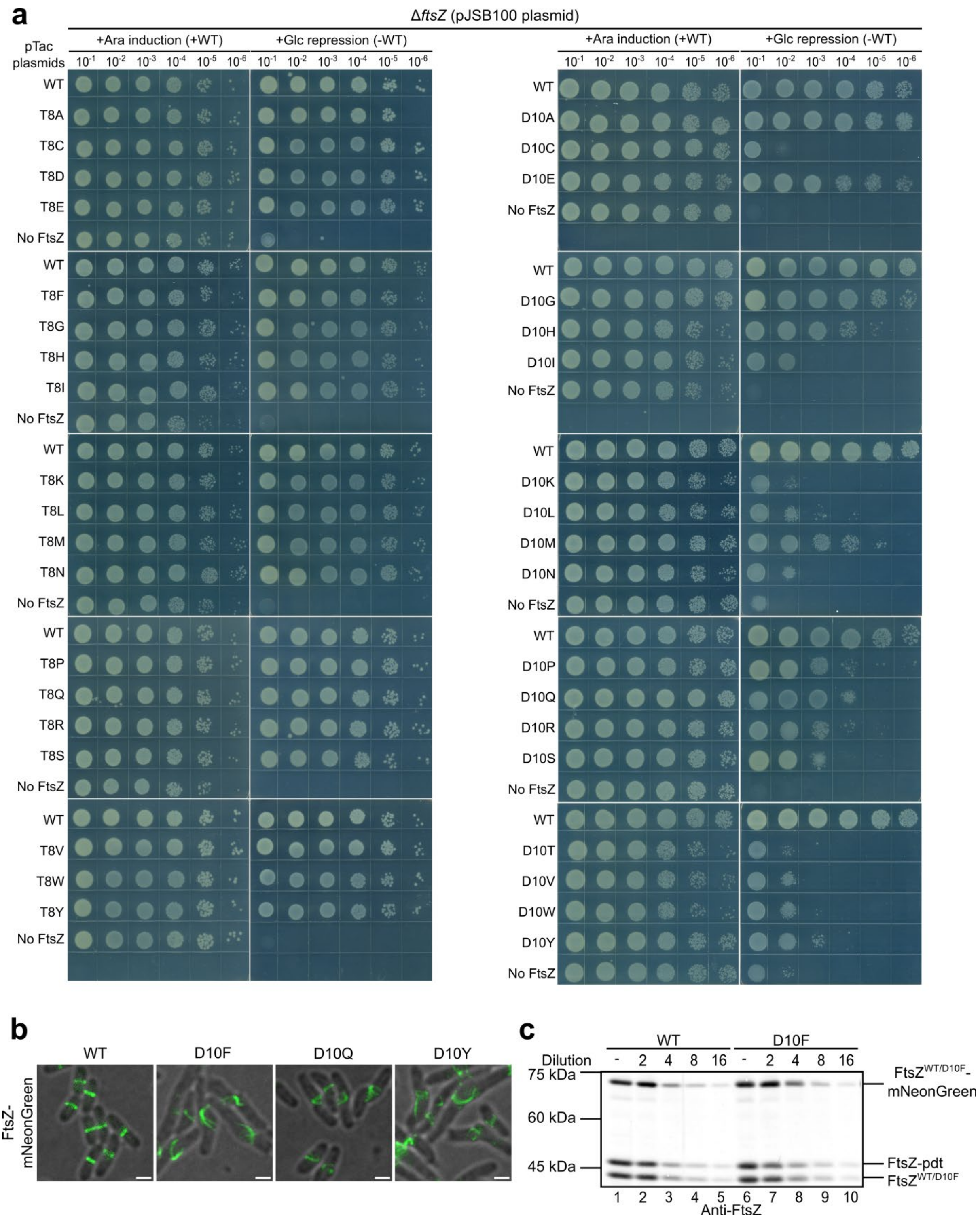

Supplementary Figure 4. Residue D10 substitution to other residues renders a functionally defective FtsZ.

a Functional complementation analyses of FtsZ variants where residue T8 (left) or D10 (right) was replaced by the indicated amino acid residues. **b** Live-cell imaging micrographs (merged brightfield and fluorescence images) showing cells expressing indicated D10 variants of FtsZ (each fused to mNeonGreen); scale bar = 1 µm. **c** Immunoblot showing that FtsZ^{D10F} was expressed at levels comparable to wild-type FtsZ. Source data are provided in the Source Data file.

Supplementary Fig. 5

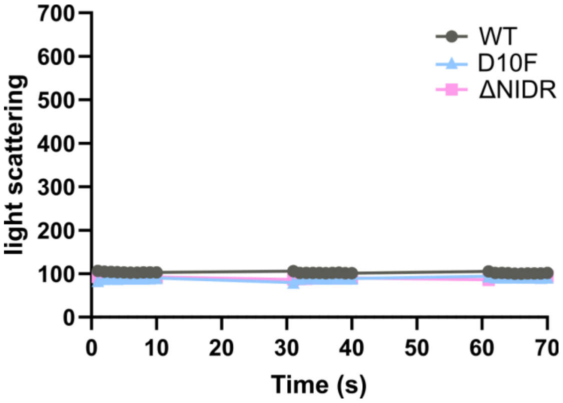

Supplementary Figure 5. Light scattering baseline curves of FtsZ^{WT}, FtsZ^{D10F} and FtsZ$^{\Delta NIDR}$ (corresponding to Fig 4a). The average scattering light baseline values for FtsZ^{WT}, FtsZ^{D10F} and FtsZ$^{\Delta NIDR}$ were 102.29, 89.516, and 91.352, respectively. These curves indicated that none of the mutants exhibited unusually high baselines compared to the WT. Source data are provided in the Source Data file.

Supplementary Fig. 6

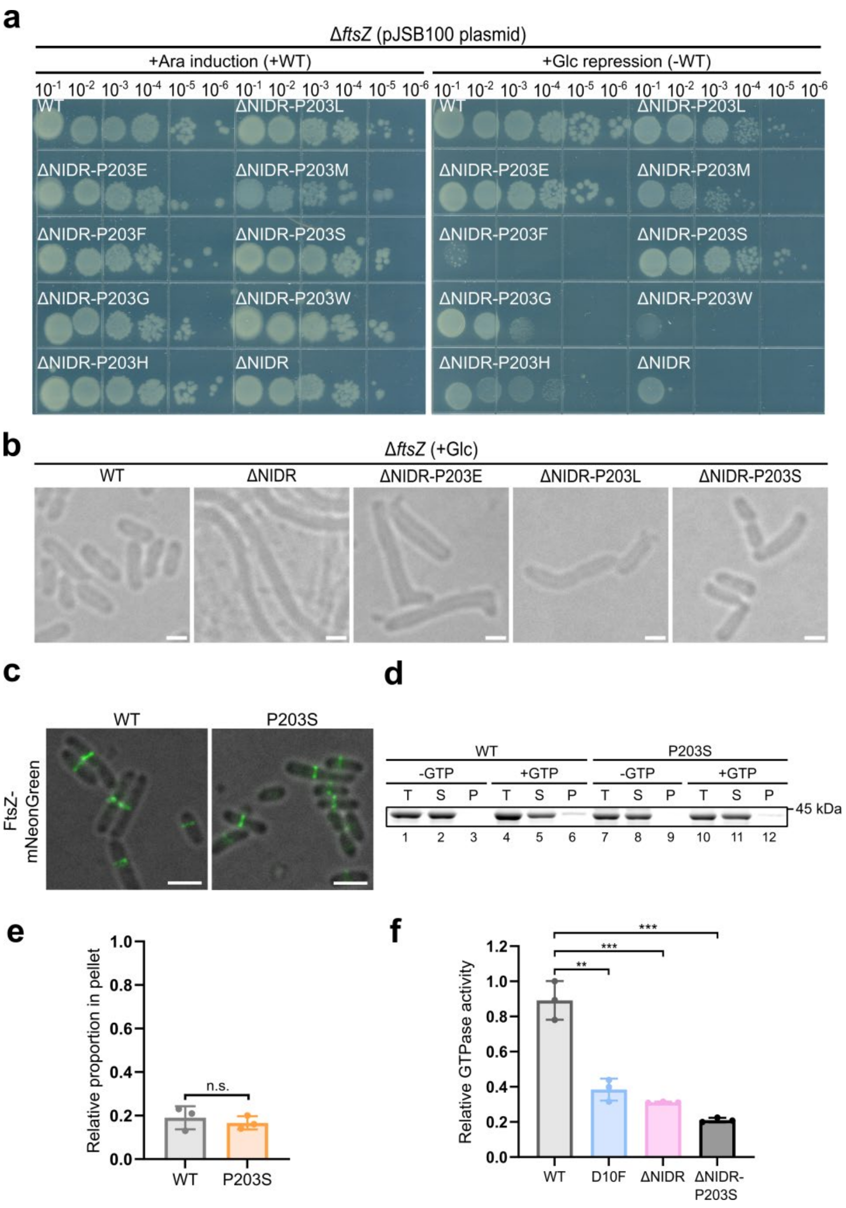

Supplementary Figure 6. P203 substitution to other amino acid residues in FtsZ$^{\Delta NIDR}$ allows the functionally defective mutant protein to largely resume its function.

a Functional complementation of FtsZ$^{\Delta NIDR}$ variants where the P203 residue was replaced by indicated amino acids. **b** Micrographs showing cells expressing indicated P203 variants derived from FtsZ$^{\Delta NIDR}$; scale bar = 1 µm. **c** Live-cell imaging micrographs (merged brightfield and fluorescence images) showing cells expressing the indicated forms of FtsZ (each fused to mNeonGreen); scale bar = 2 µm. **d** Coomassie brilliant blue staining revealed the sedimentation profile of FtsZ polymers formed from 3 µM monomeric protein in the presence of 2 mM GTP (T: total lysate; S: supernatant; P: pellet). Source data are provided in the Source Data file. **e** Quantification of FtsZ polymerization (**d**) shows no statistically significant differences in sedimented fractions (n = 3 biological replicates; data represented as the mean ± SEM; parametric unpaired two-tailed Student's *t*-test, n.s. = no significance). Source data are provided in the Source Data file. **f** Relative GTPase activity for the indicated FtsZ forms with a starting monomeric concentration of 15 µM and addition of 1 mM GTP; n = 3 biological replicates, mean ± SEM; parametric unpaired two-tailed Student's *t*-test, $^{**}P < 0.01$, $^{***}P < 0.001$; P values for the different parameters are: FtsZ^{WT}-FtsZ^{D10F}=0.0022; FtsZ^{WT}-FtsZ$^{\Delta NIDR}$=0.0008 ; FtsZ^{WT}-FtsZ$^{\Delta NIDR}$=0.0004. Source data are provided in the Source Data file.

Supplementary Fig. 7

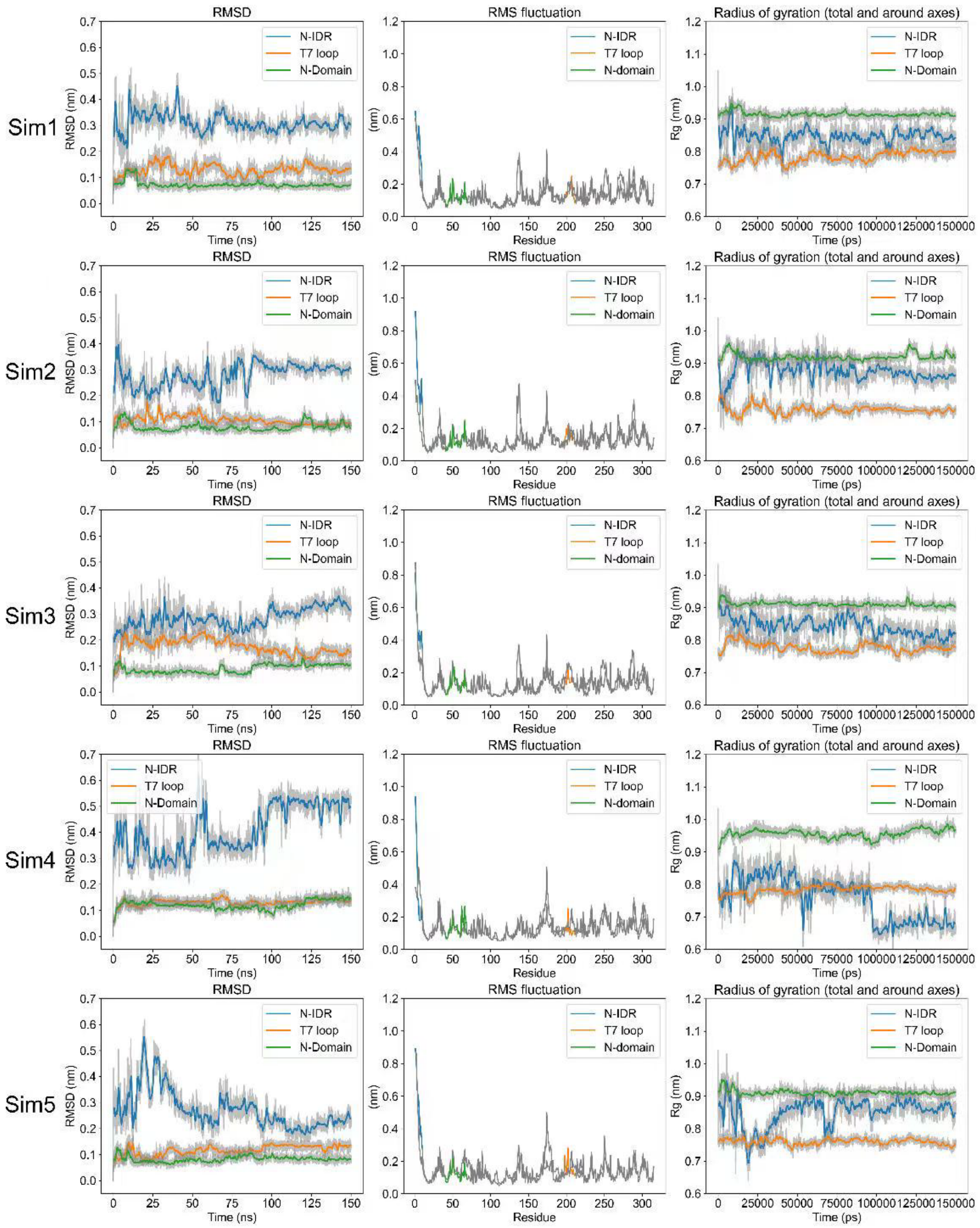

Supplementary Figure 7. Molecular dynamics simulations of the N-terminal intrinsically disordered region (N-IDR), N-domain, and T7-loop in the FtsZ dimer interface. Five independent 150 ns molecular dynamics simulations (Sim1–Sim5) were conducted at 300 K using GROMACS with the following parameters: protein FtsZ, AMBER ff99SB-ILDN force field; ligand GTP, AMBER parameters; solvent, SPC/E water model; counterions, sodium ions for system neutralization. Trajectory analysis was performed using frames sampled at 10 ps intervals. Figure panels show: (Left) Temporal evolution of root-mean-square deviation (RMSD) from initial structures, quantifying global conformational stability; (Middle) Per-residue root-mean-square fluctuation (RMSF) mapping local flexibility across the FtsZ dimer interface; (Right) Radius of gyration (Rg) trajectories tracking overall compactness changes. Color coding is consistent throughout: N-IDR (blue), T7-loop (orange), and N-domain (green). Source data are provided in the Source Data file.

Supplementary Fig. 8

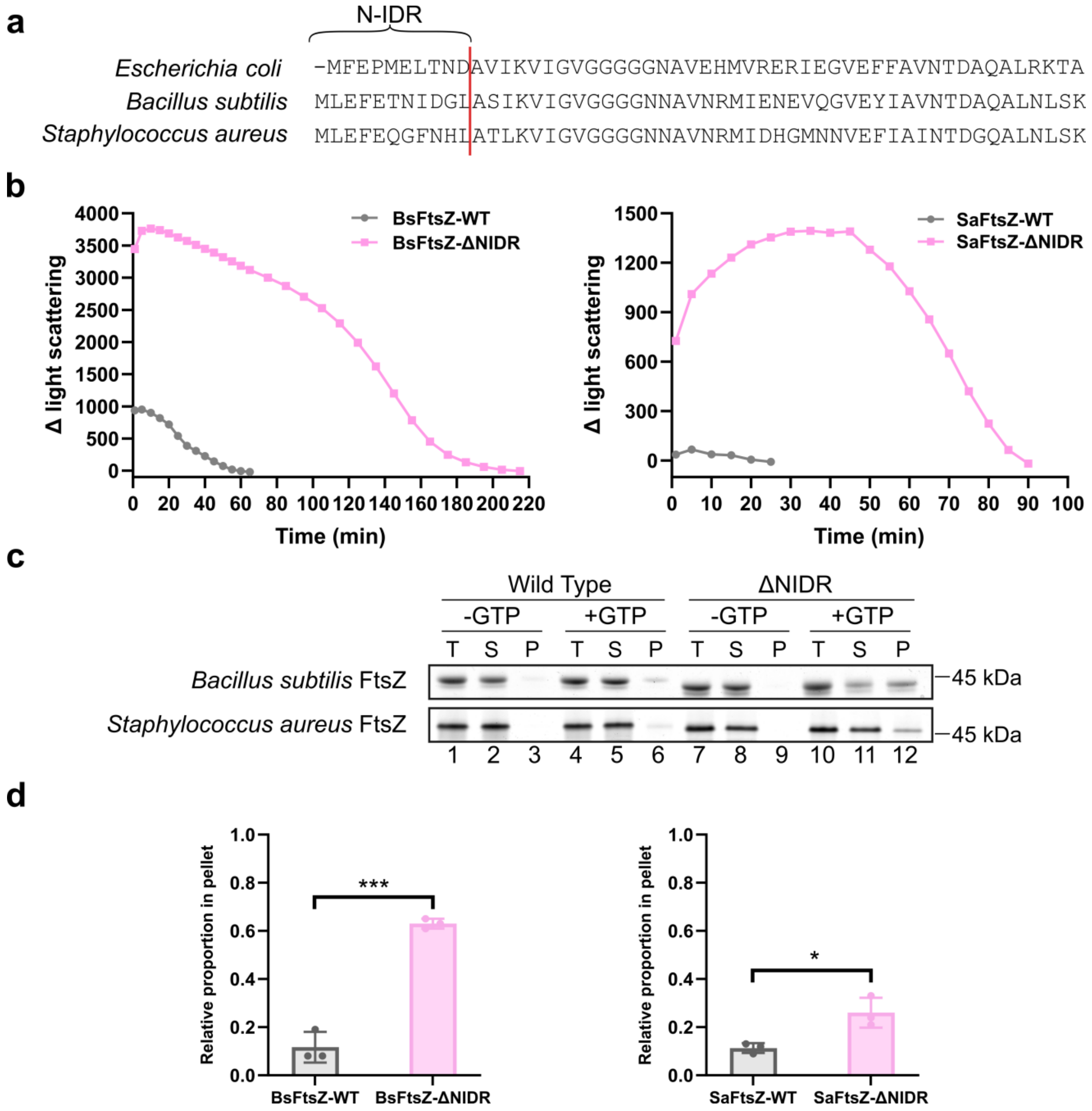

Supplementary Figure 8. The N-IDR of FtsZ derived from two other bacterial species may also act as *cis* disassembly elements. a The amino acid sequences of the FtsZ derived from indicated bacterial species showing their N-IDR sequences that were truncated to generate FtsZ$^{\Delta NIDR}$ mutants. **b** One set of representative light scattering curves of the time-dependent polymerization processes of indicated forms of FtsZ each with a starting monomeric concentration of 15 μM as initiated by 1 mM GTP and monitored by 90° angle light scattering. Source data are provided in the Source Data file. **c** Coomassie brilliant blue staining of different fractions derived from ultracentrifugation of the polymers formed by FtsZ of the indicated bacterial species; T, total; S, supernatant; P, pellet. Source data are provided in the Source Data file. **d** Relative proportions of the pellets formed by the indicated forms of FtsZ as analyzed in (**c**); n = 3 biological replicates; mean ± SEM; parametric unpaired two-tailed Student's *t*-test, ****P* < 0.001, **P* < 0.05; *P* values for the different parameters are: BsFtsZ-WT to BsFtsZ-ΔNIDR=0.0002; SaFtsZ-WT to SaFtsZ-ΔNIDR =0.0191. Source data are provided in the Source Data file.

Supplementary Table 1. The best crosslinked hit peptides for selected Bpa variants of FtsZ

Bpa-containing peptides*	Bpa replaced residues	Identified crosslinked peptides in FtsZ*	Crosslinked residues	Spectrum counts
MFEPMELTNDAVIK (1–14)	T8	(ER)IEGVEFFAVNTDAQALRK (32–51)	V42	306
		(ER)IEGVEFFAVNTDAQALRK (32–51)	A46	218
		(ER)IEGVEFFAVNTDAQALRK (32–51)	F40	114
		(ER)IEGVEFFAVNTDAQALRK (32–51)	N43	113
		(ER)IEGVEFFAVNTDAQALRK (32–51)	A48	72
		(ER)IEGVEFFAVNTDAQALRK (32–51)	D45	57
		(ER)IEGVEFFAVNTDAQALRK (32–51)	A41	56
		(ER)IEGVEFFAVNTDAQALRK (32–51)	Q47	40
		(ER)IEGVEFFAVNTDAQALRK (32–51)	T44	40
		(ER)IEGVEFFAVNTDAQALRK (32–51)	L49	17
		(ER)IEGVEFFAVNTDAQALRK (32–51)	R50	9
MFEPMELTNDAVIK (1–14)	D10	ERIEGVEFFAVNTDAQALRK (32–51)	N43	252

		(ER)IEGVEFFAVNTDAQALRK (32–51)	V42	231
		(ER)IEGVEFFAVNTDAQALRK (32–51)	A46	221
		(ER)IEGVEFFAVNTDAQALRK (32–51)	D45	94
		(ER)IEGVEFFAVNTDAQALRK (32–51)	A48	84
		(ER)IEGVEFFAVNTDAQALRK (32–51)	Q47	67
		(ER)IEGVEFFAVNTDAQALRK (32–51)	F40	57
		(ER)IEGVEFFAVNTDAQALRK (32–51)	T44	54
		(ER)IEGVEFFAVNTDAQALRK (32–51)	A41	39
		(ER)IEGVEFFAVNTDAQALRK (32–51)	R50	12
		(ER)IEGVEFFAVNTDAQALRK (32–51)	L49	11
ERIEGVEFFAVNTD AQALRK (32–51)	F40	MFEPMELTNDAVIK (1–14)	M1	268
		MFEPMELTNDAVIK (1–14)	F2	93
		MFEPMELTNDAVIK (1–14)	E3	37
KTAVGQTIQIGSGIT K (51–66)	K51	MFEPMELTNDAVIK (1–14)	E3	148
		MFEPMELTNDAVIK (1–14)	M1	72
		MFEPMELTNDAVIK (1–14)	P4	71

		MFEPMELTNDAVIK (1–14)	M5	11
		MFEPMELTNDAVIK (1–14)	F2	10
TAVQQTIQIGSGITK (52–66)	Q56	MFEPMELTNDAVIK (1–14)	M1	27
		MFEPMELTNDAVIK (1–14)	F2	27
		MFEPMELTNDAVIK (1–14)	E3	19
TAVBQTIQIGSGITK (52–66)	S62	MFEPMELTNDAVIK (1–14)	P4	137
		MFEPMELTNDAVIK (1–14)	E3	53

*** Red colored residues are those replaced by Bpa; blue colored residues are those crosslinked to corresponding, introduced Bpa residues.**

Supplementary Table 2. Longitudinal interface peptides and residues crosslinked to FtsZK51Bpa

Bpa-containing peptides*	Bpa replaced residues	Identified crosslinked peptides in FtsZ*	Crosslinked residues	Spectrum counts
KTAVGQTIQIGS GITK (51–66)	K51	GAVQGIAELITRPGLMNVDFADVR (191–214)	P203	83
		GAVQGIAELITRPGLMNVDFADVR (191–214)	G204	14
		GAVQGIAELITRPGLMNVDFADVR (191–214)	L205	12

***Red colored residues indicate those replaced by Bpa; blue colored residues indicate those crosslinked to the corresponding, introduced Bpa residue.**

Supplementary Table 3. Longitudinal interface peptides and residues crosslinked to K51Bpa of FtsZ$^{\Delta NIDR}$

Bpa-containing peptides*	Bpa replaced residues	Identified crosslinked peptides in FtsZ*	Crosslinked residues	Spectrum counts
KTAVGQTIQIGSGITK (51–66)	K51	GAVQGIAELITRPGLMNVDFA DVR (191–214)	P203	245
		GAVQGIAELITRPGLMNVDFA DVR (191–214)	G204	51
		GAVQGIAELITRPGLMNVDFA DVR (191–214)	L205	32
		GAVQGIAELITRPGLMNVDFA DVR (191–214)	N207	10
		GAVQGIAELITRPGLMNVDFA DVR (191–214)	M206	8
		GAVQGIAELITRPGLMNVDFA DVR (191–214)	V208	7

*** Red colored residues are those replaced by Bpa; blue colored residues are those crosslinked to corresponding, introduced Bpa residues.**

Supplementary Table 4. *Escherichia coli* strains used in this study

Strain	Genotype	Source/ Reference
LY928	BW25113 Δ*insH11*::aminoacyl-tRNA synthetase of Bpa-$tRNA^{Bpa}$	Author's storage
LY928-*ftsZ-pdt*	LY928 *ftsZ*::*ftsZ-pdt tag*	Author's storage
ftsZ-Avi	LY928 *ftsZ*::*ftsZ-Avi tag*	Author's storage
Δ*ftsZ*	LY928 *ftsZ::kan*^{R}	Author's storage
ftsZ-WT-mneongreen	LY928 Δ(*rhaD-rhaB*)568::*ftsZ-mneongreen*	Author's storage
ftsZ-ΔNIDR-mneongreen	LY928 Δ(*rhaD-rhaB*)568::*ftsZ-ΔNIDR-mneongreen*	Recombineering
ftsZ-D10F-mneongreen	LY928 Δ(*rhaD-rhaB*)568::*ftsZ-D10F-mneongreen*	Recombineering
ftsZ-P203S-mneongreen	LY928 Δ(rhaD-rhaB)568::*ftsZ-P203S-mneongreen*	Recombineering
ftsZ-ΔNIDR-P203S -mneongreen	LY928 Δ(*rhaD-rhaB*)568::*ftsZ-ΔNIDR-P203S-mneongreen*	Recombineering
ftsZ-WT-mscarlet	LY928 Δ(*rhaD-rhaB*)568::*ftsZ-mscarlet*	Recombineering
ftsZ-ΔNIDR-mscarlet	LY928 Δ(*rhaD-rhaB*)568::*ftsZ-ΔNIDR- mscarlet*	Recombineering
ftsZ-D10F-mscarlet	LY928 Δ(*rhaD-rhaB*)568::*ftsZ-D10F-mscarlet*	Recombineering
*Trans*10	F^- *mcr*A Δ(*mrr-hsd*RMS-*mcr*BC) φ80 *lac*ZΔM15Δ*lac*X74 *rec*A1 *ara*Δ139Δ(*ara-leu*)7697 *gal*U *gal*K *rps*L (Str^R)*end*AI *nup*G	Author's storage
BL21(DE3)	F^- *omp*T *hsd*S_B(r_B^- m_B^-) *gal dcm*(DE3)	Author's storage

Supplementary Table 5. Plasmids used in this study

Plasmid	Genotype[a]	ori	Reference/Source
pTet-FtsZ-WT	*bla* P_{tet1}::*ftsZ*	pBR322	Author's storage
pTac-FtsZ-WT	*bla* $P_{T\text{-}23105}$::*ftsZ*	pBR322	This study
pTet-FtsZ-Bpa-mutants	*bla* P_{tet1}::*ftsZ-Bpa-mutants*	pBR322	This study
pTac-FtsZ-Bpa-mutants	*bla* $P_{T\text{-}23105}$::*ftsZ-Bpa-mutants*	pBR322	This study
pYR5C-tetR	*cat* P_{23116}::*tetR*	p15A	Author's storage
pTet-FtsZ-Bpa-His	*bla* P_{tet1}::*ftsZ-Bpa-his tag*	pBR322	This study
pTac-FtsZ-ΔNIDR	*bla* $P_{T\text{-}23105}$::*ftsZ-ΔNIDR*	pBR322	This study
pTac-FtsZ-D10mutants	*bla* $P_{T\text{-}23105}$::*ftsZ-D10mutants*	pBR322	This study
pTac-FtsZ-P203S	*bla* $P_{T\text{-}23105}$::*ftsZ-P203S*	pBR322	This study
pJSB100	*cat* P_{ara}::*ftsZ*	pBR322	(*1*)
pTac-FtsA-mNeonGreen	*bla* P_{23117}::*ftsA-mneongreen*	pBR322	Author's storage
pYLC-rha-mNeonGreen-FtsN	*bla* P_{rha}::*mneongreen-ftsN*	pBR322	This study
pYRG-MinC	*gen* P_{tet1}::*minC*	cdf	This study
pET28a-FtsZ-WT	*kan* $P_{T7\text{-}lacO}$::*ftsZ-WT*	pBR322	This study
pET28a-FtsZ-ΔNIDR	*kan* $P_{T7\text{-}lacO}$::*ftsZ-ΔNIDR*	pBR322	This study
pET28a-FtsZ-D10F	*kan* $P_{T7\text{-}lacO}$::*ftsZ-D10F*	pBR322	This study
pET28a-FtsZ-Bpa-mutants	*kan* $P_{T7\text{-}lacO}$::*ftsZ-Bpa-mutants*	pBR322	This study
pBAD-BpaRS-tRNA^{Bpa}	*bla* P_{BAD}::MjBpaRS-tRNA^{Bpa}	pBR322	Author's storage
pET28a-MinC-His	*kan* $P_{T7\text{-}lacO}$::*minC-his*	pBR322	This study
pYR6C-*mf*-Lon	*cat* P_{tet}::*mf-lon*	p15A	Author's storage

pTac-FtsZ-ΔNIDR-P203mutants	*bla* $P_{T\text{-}23105}$::*ftsZ-ΔNIDR-P203mutants*	pBR322	This study
pET28a-FtsZ-ΔNIDR-P203S	*kan* $P_{T7\text{-}lacO}$::*ftsZ-ΔNIDR-P203S*	pBR322	This study
pET28a-FtsZ-P203S	*kan* $P_{T7\text{-}lacO}$::*ftsZ-P203S*	pBR322	This study
pET28a-BsFtsZ-WT	*kan* $P_{T7\text{-}lacO}$::*ftsZ-BsWT*	pBR322	This study
pET28a-BsFtsZ-ΔNIDR	*kan* $P_{T7\text{-}lacO}$::*ftsZ-BsΔNIDR*	pBR322	This study
pET28a-SaFtsZ-WT	*kan* $P_{T7\text{-}lacO}$::*ftsZ-SaWT*	pBR322	This study
pET28a-SaFtsZ-ΔNIDR	*kan* $P_{T7\text{-}lacO}$::*ftsZ-SaΔNIDR*	pBR322	This study

[a]P_{ara} and P_{BAD} are promoters induced by arabinose; P_{rha}, $P_{T7\text{-}lacO}$, and P_{tet} are promoters induced by rhamnose, isopropyl-1-thio-*β*-D-galactopyranoside and anhydrotetracycline, respectively. P_{23116}, P_{23117} are synthetic constitutive promoters (selected from the Anderson promoter collection: parts.igem.org/Promoters/Catalog/E.coli/Constitutive). P_{tet1} is a promoter from which a protein is expressed in a leaky manner. $P_{T\text{-}23105}$ represents a synthetic constitutive promoter P_{23105} with the *λ*t1 transcriptional terminator and *tetO* sequence added in the front and middle of it to achieve suppressed or constant expression with or without TetR and anhydrotetracycline.

Supplementary Table 6. Details of Molecular Dynamic simulation systems.

Parameter	Value
System	EcFtsZ (1–315) dimer
Dimensions	130.6 Å x 130.6 Å x 130.6 Å
No. atoms	218960
No. water	69890
No. Sodium ions	46, neutralize net charge on the protein and ligand
No. GTP	2
Duration	150 ns

Supplementary References

1. Stricker, J. & Erickson, H. P. *In vivo* Characterization of *Escherichia coli ftsZ* mutants: effects on Z-ring structure and function. *J. Bacteriol.* **185**, 4796–4805 (2003).
