# Supplementary material for: A FtsZ cis disassembly element acts in Z-ring assembly during bacterial cell division
Source: Nat Commun. 2025 Jun 4;16:5194. doi: 10.1038/s41467-025-60517-7 (PMC12137876; doi:10.1038/s41467-025-60517-7)
Supplement: Supplementary file 2 — Description of Additional Supplementary Files [file 41467_2025_60517_MOESM2_ESM.pdf]

Description of Additional Supplementary Files:

Supplementary Data 1: primers used in this study
