# Supplementary material for: A FtsZ cis disassembly element acts in Z-ring assembly during bacterial cell division
Source: Nat Commun. 2025 Jun 4;16:5194. doi: 10.1038/s41467-025-60517-7 (PMC12137876; doi:10.1038/s41467-025-60517-7)
Supplement: Supplementary file 4 — Reporting Summary [file 41467_2025_60517_MOESM4_ESM.pdf]

nature portfolio

Corresponding author(s): Zengyi Chang

Last updated by author(s): May 14, 2025

Reporting Summary

Nature Portfolio wishes to improve the reproducibility of the work that we publish. This form provides structure for consistency and transparency in reporting. For further information on Nature Portfolio policies, see our Editorial Policies and the Editorial Policy Checklist.

Statistics

For all statistical analyses, confirm that the following items are present in the figure legend, table legend, main text, or Methods section.

n/a	Confirmed	
[ ]	[x]	The exact sample size (*n*) for each experimental group/condition, given as a discrete number and unit of measurement
[ ]	[x]	A statement on whether measurements were taken from distinct samples or whether the same sample was measured repeatedly
[ ]	[x]	The statistical test(s) used AND whether they are one- or two-sided<br>*Only common tests should be described solely by name; describe more complex techniques in the Methods section.*
[x]	[ ]	A description of all covariates tested
[x]	[ ]	A description of any assumptions or corrections, such as tests of normality and adjustment for multiple comparisons
[ ]	[x]	A full description of the statistical parameters including central tendency (e.g. means) or other basic estimates (e.g. regression coefficient) AND variation (e.g. standard deviation) or associated estimates of uncertainty (e.g. confidence intervals)
[ ]	[x]	For null hypothesis testing, the test statistic (e.g. *F*, *t*, *r*) with confidence intervals, effect sizes, degrees of freedom and *P* value noted<br>*Give P values as exact values whenever suitable.*
[x]	[ ]	For Bayesian analysis, information on the choice of priors and Markov chain Monte Carlo settings
[x]	[ ]	For hierarchical and complex designs, identification of the appropriate level for tests and full reporting of outcomes
[x]	[ ]	Estimates of effect sizes (e.g. Cohen's *d*, Pearson's *r*), indicating how they were calculated

Our web collection on statistics for biologists contains articles on many of the points above.

Software and code

Policy information about availability of computer code

Data collection	Data collected using NIS-Elements AR (version 5.42.00) , MetaMorph (v7.10.4.407) or Invitrogen EVOS FL Auto 2. Molecular dynamics simulations were conducted using the GROMACS 2024.2 package. Protein structure was modeled using the SWISS-MODEL server. Topology and force field parameters of GTP molecule were generated using the ACPYPE tool.
Data analysis	Micrography image analysed using Fiji(v1.48) with open-source plugins Image stabilizer, FRAP profiler V2 and KymographBuilder. Protein bands processed using GNU image manipulation (v2.10). Mass spectrometry analysed using pLink (v2.3.9). Statistical analysed using GraphPad Prism (v10.1). Molecular dynamics simulations data analysed using the GROMACS tools (mdstat, rms for RMSD, rmsf for RMSF, and gyrate for Rg), the visualization using the PyMol software (2.5) and Matplotlib (v3.6.3).

For manuscripts utilizing custom algorithms or software that are central to the research but not yet described in published literature, software must be made available to editors and reviewers. We strongly encourage code deposition in a community repository (e.g. GitHub). See the Nature Portfolio guidelines for submitting code & software for further information.

Data

Policy information about availability of data

All manuscripts must include a data availability statement. This statement should provide the following information, where applicable:

- Accession codes, unique identifiers, or web links for publicly available datasets
- A description of any restrictions on data availability
- For clinical datasets or third party data, please ensure that the statement adheres to our policy

Simulation data generated in this study were deposited in the Zenodo repository (accession code: 15186509; http://zenodo.org/records/15186509). Mass spectrometry data generated in this study have been deposited in the ProteomeXchange Consortium through the PRIDE partner repository with dataset identifier PXD063736 (http://www.ebi.ac.uk/archive/projects/PXD063736). Source data are provided with this paper.

Research involving human participants, their data, or biological material

Policy information about studies with human participants or human data. See also policy information about sex, gender (identity/presentation), and sexual orientation and race, ethnicity and racism.

Reporting on sex and gender	N/A
Reporting on race, ethnicity, or other socially relevant groupings	N/A
Population characteristics	N/A
Recruitment	N/A
Ethics oversight	N/A

Note that full information on the approval of the study protocol must also be provided in the manuscript.

Field-specific reporting

Please select the one below that is the best fit for your research. If you are not sure, read the appropriate sections before making your selection.

☒ Life sciences ☐ Behavioural & social sciences ☐ Ecological, evolutionary & environmental sciences

For a reference copy of the document with all sections, see nature.com/documents/nr-reporting-summary-flat.pdf

Life sciences study design

All studies must disclose on these points even when the disclosure is negative.

Sample size	The sample size for all experiments (n) is indicated in the figure legends.
Data exclusions	No data exclusion was performed.
Replication	All experiments were conducted as multiple, independent replicates as indicated in the figure legends and Methods.
Randomization	Allocating experimental groups is not relevant to this study.
Blinding	Blinding is not relevant to this study.

Reporting for specific materials, systems and methods

We require information from authors about some types of materials, experimental systems and methods used in many studies. Here, indicate whether each material, system or method listed is relevant to your study. If you are not sure if a list item applies to your research, read the appropriate section before selecting a response.

Materials & experimental systems

n/a	Involved in the study
☐	☒ Antibodies
☒	☐ Eukaryotic cell lines
☒	☐ Palaeontology and archaeology
☒	☐ Animals and other organisms
☒	☐ Clinical data
☒	☐ Dual use research of concern
☒	☐ Plants

Methods

n/a	Involved in the study
☒	☐ ChIP-seq
☒	☐ Flow cytometry
☒	☐ MRI-based neuroimaging

Antibodies

Antibodies used	Anti-FtsZ antibodies (Rabibit; self-made); Goat anti-rabbit IgG (H+L), IDRye 800CW (Bioss, Beijing, bs-40295G-IDRye8); Streptavidin, Alexa Fluor 680 (Bioss, Beijing, bs-0437P-AF680)
Validation	Anti-FtsZ antibodies were validated in previous literatures (https://doi.org/10.7554/eLife.35578;https://doi.org/10.1038/s41421-019-0080-3). Goat anti-rabbit IgG(H+L), IDRye 800CW was confirmed by the manufacturer (https://www.biosschina.com/#/productDetail?goods_id=19472). Streptavidin Alexa Fluor 680 was confirmed by the manufacturer (https://www.bioss.com.cn/upload/datasheet/bs-0437P-AF680_201.pdf).

Plants

Seed stocks	N/A
Novel plant genotypes	N/A
Authentication	N/A
